# Ancient Chinese Medicine Herbal Formula Huanglian Jiedu Decoction as a Neoadjuvant Treatment of Chemotherapy by Improving Diarrhea and Tumor Response

**DOI:** 10.3389/fphar.2020.00252

**Published:** 2020-03-10

**Authors:** Yau-Tuen Chan, Fan Cheung, Cheng Zhang, Bowen Fu, Hor-Yue Tan, Hisayoshi Norimoto, Ning Wang, Yibin Feng

**Affiliations:** ^1^School of Chinese Medicine, Li Ka Shing Faculty of Medicine, The University of Hong Kong, Pokfulam, Hong Kong; ^2^PuraPharm International (H.K.) Ltd., Shatin, Hong Kong

**Keywords:** Huanglian Jiedu decoction, chemotherapy-induced diarrhea, 5-fluorouracil, irinotecan, tumor response

## Abstract

**Background:**

Diarrhea is a major gastrointestinal complication in cancer patients receiving chemotherapy. Prognosis and treatment of chemotherapy-induced diarrhea (CID) remain unsatisfactory. This study aims to explore the potential of an ancient Chinese Medicine herbal formula Huanglian Jiedu Decoction (HLJDD) as an adjuvant treatment on CID.

**Method:**

HLJDD extract was prepared by GMP manufacturing standard with quality and stability being checked. 5-fluorouracil (5-Fu) and irinotecan (CPT-11)-induced diarrhea model in mice was established and pre-, co- and post-treatment of HLJDD was implemented. Mechanism of action was explored by detecting related protein expression. In addition, the effect of HLJDD on diarrhea and tumor response induced by clinical regimens FOLFOX and FOLFIRI was measured in murine orthotopic colorectal cancer model.

**Results:**

HLJDD exhibited consistency in quality and stability after 24-month storage. Pre-treatment of HLJDD, but not co-treatment or post-treatment, could significantly improve the diarrhea score, body weight loss and intestinal damage in 5-Fu- and CPT-11-treated mice. Pre-treatment of HLJDD reduced cell apoptosis in the intestine of chemotherapy-treated mice, and promoted renewal of intestinal cell wall. CD44 was predicted as the potential target of HLJDD-containing compounds in CID. HLJDD pre-treatment induced presentation of CD44-postive cells in the intestine of chemotherapy-treated mice, and initiated expression of stemness-associated genes. Transcriptional products of the downstream Wnt signaling of CD44 were elevated. Furthermore, pre-treatment of HLJDD could significantly improve the tumor response of clinical chemotherapy regimens FOLFOX and FOLFIRI in orthotopic colorectal cancer, and reduce diarrhea and intestinal damage. Conclusion: Our study suggests the potential of HLJDD as a neoadjuvant treatment of chemotherapy by reducing diarrhea and improving tumor response.

## Introduction

Cancer chemotherapy is one of the most common non-surgical cancer therapeutic approaches (Mishra et al., 2018; Ribas and Wolchok, 2018; Levine and Kroemer, 2019; Tang et al., 2019). It works specifically by damaging the cancer cells or slowing down their growth, yet it is accompanied with patient discontinuation problem due to the associated adverse events and drug resistance. Highly proliferative tissues such as gastrointestinal mucosa, skin and hematopoietic system may be the non-specific targets of chemotherapeutic agents leading to early stage of toxicities, and late adverse effects referring to the low proliferating tissues which further leads to fibrosis, neuronal, vascular and respiratory damage (Di Fiore and Van Cutsem, 2009). Gastrointestinal toxicity is often reported when the chemotherapy disrupts the colon permeability and intestinal mucosal structure. The most common symptoms of chemotherapy induced GI toxicity are diarrhea, vomiting, anorexia, and nausea (Mitchell, 2006). Chemotherapy induced diarrhea (CID) is a result of intestinal mucosa damage, which repeatedly triggers apoptotic and inflammatory events in intestinal epithelium and bowel wall. CID may be life threatening due to the continuous loss of electrolytes and fluids accompanied with malnutrition (Stein et al., 2010).

Accumulating studies have demonstrated that Chinese herbal medicines could be adjunct therapy for cancer patients, predominantly in reducing cancer therapy-associated adverse effects and thereby improving life quality (Chen et al., 2016; Gou et al., 2016). A recent double-blinded randomized study demonstrated that intervention of Chinese herbal medicine for breast or colon cancer patients significantly reduced chemotherapy associated GI toxicity, nausea in particular (Mok et al., 2007). Another Chinese herbal formulation, PHY906 which employed by practitioners for GI complications, showed promising outcome in reducing irinotecan induced GI toxicity. Previous clinical studies have shown intervention by PHY906 reduced the incidence of CPT-11 associated diarrhea (Kummar et al., 2011) and phase II clinical trial is currently conducted at U.S. on metastatic colorectal cancer patients who undergoing chemotherapy (Health, 2019). As opposed to prevention of the intestinal damage incurred by CPT-11, PHY906 recovers the functions of intestinal cells through increased progenitor cells regeneration and blocked CPT-11 induced inflammation (Lam et al., 2010).

HLJDD is composed of four Chinese herbal species, which includes *Coptis chinensis* Franch, *Phellodendron amurense* Rupr, *Gardenia jasminoides* J. Ellis and *Scutellaria baicalensis* Georgi and constructed by a total of 29 major single constituents. It has been reported to associate with various pharmacological activities such as arthritis, type II diabetes and ischemic stroke (Hu et al., 2013; Wang et al., 2013; Zhang et al., 2014). Our recent work has also demonstrated the inhibitory effect of HLJDD in cancer growth and angiogenesis in xenograft murine model through inactivation of eEF2 activity (Wang et al., 2015). Furthermore, previous studies postulated that HLJDD exerts protective effect on indomethacin-triggered intestinal damage by reducing inflammation related adenosine deaminase activity (Watanabe-Fukuda et al., 2009). In this study, we for the first time evaluated the possibility of using HLJDD for the treatment of CID. We chose 5-fluororacial (5-Fu) and irinotecan (CPT-11), which were commonly used as first-line chemotherapy in cancer treatment but were frequently reported to induce diarrhea in patients, to establish the animal model of CID. Effects on different treatments of HLJDD were systemically evaluated while its mechanism of action was proposed by computational prediction and validated by experimental approaches. Notably, the combination of HLJDD with clinical used chemotherapy regimens to improve tumor regression and reduce CID was studied in an orthotopic colorectal cancer model.

## Materials and Methods

### Chemicals, Reagents and Antibodies

5-Fu (Sigma-Aldrich, United States), oxaliplatin (Selleckchem, United States), Folinic acid (Sigma-Aldrich, United States), CPT-11 (Selleckchem, United States) and loperamide were obtained by purchase.

### Preparation of HLJDD and Quality Control

Preparation of HLJDD was performed in Good Manufacturing Practice according to our previous studies (Wang et al., 2015). 0.1 g powdered extract of HLJDD was precisely weighed by analytical balance, and then dissolved in 10 mL 50% Methanol-H_2_O. Samples were then sonicated for 30 min to get completely dissolved followed by centrifugation at 4,000 rpm for 10 min. 1 ml of supernatant was filtered with 0.45 μm filter. Preparation was conducted in triplicate. 2.0 mg of reference chemicals, including berberine, baicalin and geniposide, were precisely weighed and dissolved in 10 mL 50% Methanol-H_2_O. Samples and reference chemicals were analyzed with rapid separation liquid chromatography-combined diode array detector (RSLC-DAD, UltiMate^TM^ 3000, Thermofisher, United States). Separation was performed on C18 HPLC/UHPLC column provided by ACE (100 × 2.1 mm) with mobile phase shown in Table 1. The flow rate is 0.5 mL/min. The amounts of berberine, baicalin, phellodendrine and geniposide in HLJDD extracts at 1-, 3-, 6-, 12-, and 24-storage were quantified.

**TABLE 1:** Chromatography separation program.

Time (min)	Acetonitrile (%)	0.05% KH_2_PO_4_, 0.05% TEA in H_2_O, pH2.5 (%)
0	5	95
1	5	95
3	6	94
7	6	94
10	10	90
15	15	85
19	15	85
20	16	84
25	16	84
28	23	77
35	65	35

### Chemotherapy-Induced Diarrhea Model in Mice

Study protocol has been approved by the Committee on the Use of Live Animals in Teaching and Research (CULATR) of the University of Hong Kong (Ref. No: 4433-17). Chemotherapeutic treatment was designed to closely imitate the clinical regimens. To establish the 5-Fu-induced CID model, mice were given intraperitoneal injection of 50 mg/kg 5-Fu solution for the first 4 days and had a 3-day rest. The 5-Fu treatment cycle was repeated once. To establish the CPT-11-induced CID model, mice were given intraperitoneal injection of 125 mg/kg CPT-11 solution for the first 3 days and had a 4-day rest. The CPT-11 treatment cycle was repeated once. In both models, pre-, co-, and post-treatment of HLJDD would be tested. In each treatment, daily oral gavages of 50, 100, 200 mg/kg HLJDD for 2 weeks were tested (Figures 2A, 3A).

**FIGURE 1:**
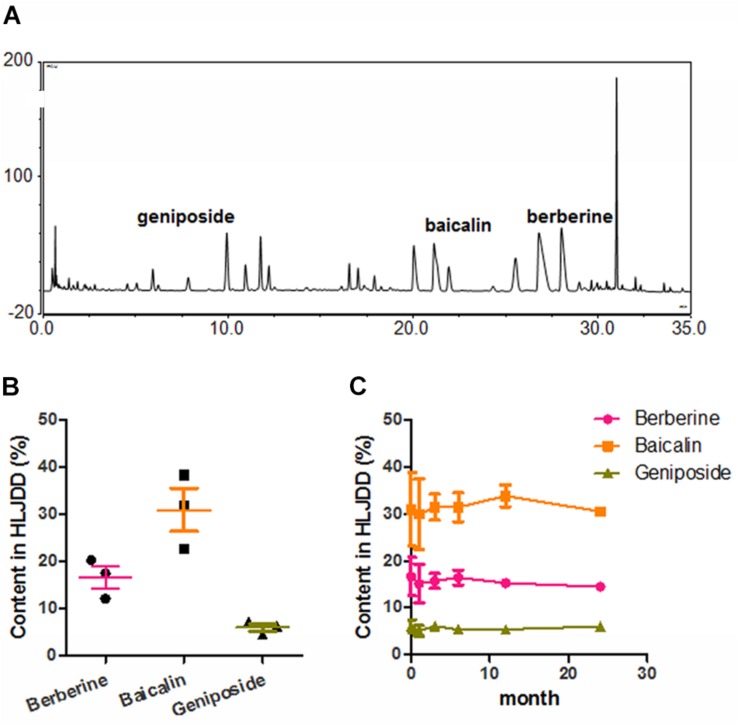
Quality control of HLJDD. **(A)** Showed the representative chromatogram of HLJDD extracts. Three major compounds, berberine, baicalin and geniposide, were identified on the chromatogram by matching the retention time and UV spectrum with those of reference compounds; **(B)** showed the inter-batches quality of HLJDD as represented by the relative contents of berberine, baicalin and geniposide; **(C)** showed that stability of HLJDD over 24-month storage as represented by the relative contents of berberine, baicalin, and geniposide.

**FIGURE 2:**
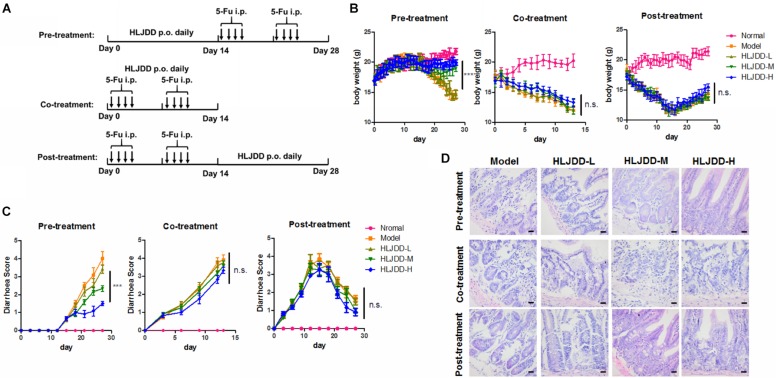
Pre-treatment but not co-treatment or post-treatment of HLJDD improved 5-Fu-induced diarrhea. **(A)** Showed the treatment scheme; **(B)** showed that pre-treatment but not co-treatment or post-treatment of HLJDD improved body weight loss induced by 5-Fu; **(C)** showed that pre-treatment but not co-treatment or post-treatment of HLJDD improved diarrhea score induced by 5-Fu; **(D)** showed that pre-treatment but not co-treatment or post-treatment of HLJDD improved the histological structure of jejunum damaged by 5-Fu. ***p < 0.001 when compared with the model group.

**FIGURE 3:**
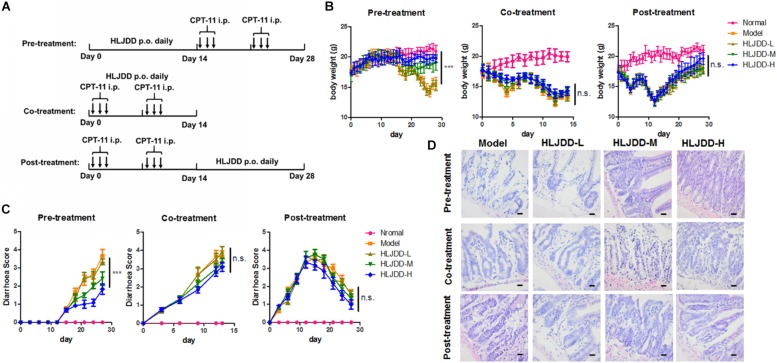
Pre-treatment but not co-treatment or post-treatment of HLJDD improved CPT-11-induced diarrhea. **(A)** Showed the treatment scheme; **(B)** showed that pre-treatment but not co-treatment or post-treatment of HLJDD improved body weight loss induced by CPT-11; **(C)** showed that pre-treatment but not co-treatment or post-treatment of HLJDD improved diarrhea score induced by CPT-11; **(D)** showed that pre-treatment but not co-treatment or post-treatment of HLJDD improved the histological structure of jejunum damaged by CPT-11. ***p < 0.001 when compared with the model group.

### Orthotopic Colorectal Cancer Model in Mice

Study protocol has been approved by the CULATR of the University of Hong Kong (Ref. No.: 4505-17). The orthotopic implantation of colorectal cancer mice model was established according to literature report (Tseng et al., 2007). In brief, colon cancer cell HT-29 transfected with luciferase reporter was subcutaneously injected to the left flank of athymic nude mice. When the cells formed around 1-cm^3^ tumors, mice were sacrificed by overdose of pentobarbital (200 mg/kg, intraperitoneally). Tumor was dissected out and cut into 2-mm^3^ pieces. A small cube of tumor was then implanted into the cecum of mice. One week after implantation, mice were screened under live animal imager to confirm formation of orthotopic tumor. Qualified mice were randomized and given treatment for 4 weeks. Mice received either 200 mg/kg/day HLJDD or same volume of water via oral gavage for the first weeks, then were given FOLFOX or FOLFIRI chemotherapy regimens for another 2 weeks. Treatment frequency and doses of the chemotherapeutic regimen were set accordingly to the clinical usage and literature report of the particular regimen. Loperamide in the positive control group was given during chemotherapy.

### Measurement of Diarrhea and Intestinal Damage

A grading system for diarrhea will be used to score the diarrhea severity every 3 days. The scoring system can be interpreted as follows, (0) (no diarrhea): solid stool with no sign of soiling around the anus. The stool is very firm when subjected to pressure with tweezers; (1) (very mild diarrhea): formed stools that appear moist on the outside, and no sign of soiling around the anus. Stool is less firm when considerable pressure applied with tweezers; (2) (mild diarrhea): formed stools that appear moist on the outside, and some signs of soiling around anus. Stools will easily submit to pressure applied with tweezers; (3) (diarrhea): no formed stools with a mucous-like appearance. Considerable soiling around the anus and the fur around tail. Mouse takes a long time to pass stool; (4) (severe, watery diarrhea): mostly clear or mucous-like liquid stool with very minimal solid present and considerable soiling around anus (Pearson et al., 2013).

1.0 cm samples of jejunum will be collected and fixed in 4% paraformaldehyde. And samples will be processed for hematoxylin-eosin (H&E) staining and analyzed by two independent trained histologists. Patho-morphological changes in intestinal sections will be scored as: 4: Severe; 3: Markedly abnormal; 2: Moderate; 1: Mild; or 0: Normal (Kojouharov et al., 2014).

### Immunofluroscence

Paraffin-embedded sections were rehydrated and antigens on the sections were retrieved with citric buffer (10 mM Sodium Citrate, 0.05% Tween 20, pH 6.0) via at 100°C for 5 min. After washing, the tissues were blocked with 10% normal goat serum in PBS supplemented with 0.5% Tween 20. Primary antibodies (1:100 v/v dilution in blocking buffer) were then applied and incubated overnight at 4°C. Then appropriate secondary antibodies were added. Expression of related protein targets were then examined under confocal microscope (LSM780, Carl Zeiss, Germany). 4′,6-diamidino-2-phenylindole (DAPI) was used to stain the cell nuclei. TUNEL assay was performed with ApoBrdU DNA Fragmentation Assay (Biovision, United States).

### Quantitative Real-Time PCR

Total RNA from tissue was extracted with Trizol method (Invitrogen, United States). cDNA was prepared with first strand synthesis kit (Takara, Japan). Quantitative real-time PCR was performed with SYBR master mix (Takara, Japan) on the LC480 platform (Roche, United States). Primers used in this study were listed in Table 2.

**TABLE 2:** Primer list.

Gene	Forward primer	Reverse primer
Lgr5	TCTTCACCTCCTACC TGGACCT	GGCGTAGTCTGCTATG TGGTGT
Olfm4	CCAGCTGGAGGTGG AGATAA	GCTGATGTTCACCA CACCAC
Wnt3	CAAGCACAACAATGA AGCAGG	TCGGGACTCACGGTG TTTCTC
Axin2	GAGTAGCGCCGTGTTA GTGACT	CCAGGAAAGTCCGGAAGA GGTATG
Fzd5	CTTGTTTCCAAAGTCCAAT CAAGTG	GCCTACTCTTCACCCTTC TTTAACG
Pygo2	GTTTGGGCTGTCCTGAA AGTCTG	ATAAGGGCGCCGAA AGTTGA

### Data Mining

The interaction between components from HLJDD and protein targets was retrieved from the Comparative Toxicogenomics Database (CTD^1^). Meanwhile, the sequencing data in Gene Expression Omnibus (GEO) database GSE28873 and GSE11722 was accessed to retrieved genes of intestine cells being regulated commonly by 5-Fu and CPT-11. Venn analysis was performed to gain a group of a common gene of the three lists^2^.

### Statistical Analysis

Data was present as Mean ± SD. Differences were measured with an ordinary two-way ANOVA with LSD multiple comparisons, with *p* < 0.05 was considered statistically significant.

## Results

### HLJDD Is Consistent in Quality and Suitable for Long-Time Storage

To ensure the quality of HLJDD used in this study, we established a chromatographic fingerprint (Figure 1A) of the formula. Three major components, berberine from Coptidis Rhizoma and Phellodendri Chinensis Cortex., baicalin from Scutellaria baicalensis Georgi and geniposide from Gardenia jasminoides Ellis, were selected as chemical markers for the quality control. We first of all tested the inter-batch consistence of HLJDD produced by GMP manufacturer via quantifying berberine, baicalin and geniposide in three batches of the extracts, and found no significant difference in amount of each compound among three batches of HLJDD (Figure 1B). Then the stability of HLJDD kept at ordinary indoor environment for 1, 3, 6, 12, and 24 months was measured. The amounts of berberine, baicalin and geniposide remained unchanged after 24-month storage (Figure 1C). These data suggest that production of HLJDD in GMP manufacturing is reproducible in batches and the extracts are suitable for up to 2 years storage under ordinary indoor condition.

### Pre-treatment of HLJDD Improved 5-Fu-Induced Diarrhea and in Mice

HLJDD has been reported to possess various biological activities and was proposed to potentially treat diseases such as diabetes (Chen et al., 2018), hepatic damage (Wei et al., 2018), ischemic stroke (Zhang et al., 2017), and cancer (Wang et al., 2015). To determine if oral administration of HLJDD can relieve CID, we established 5-Fu-induced intestine damage model with some modification on previous published protocol (Sakai et al., 2013). Pre-treatment, co-treatment and post-treatment of HLJDD with 5-Fu were tested and the scheme was shown in Figure 2A. Significant reduction in body weight of mice treated with 5-Fu was observed. We also observed a significant increase of diarrhea. Two-week pre-treatment of HLJDD potently prevented 5-Fu-induced body weight loss as well as diarrhea in mice in a dose-dependent manner, and 200 mg/kg HLJDD pretreatment for 2 weeks showed optimal effect. The diarrhea scores dropped to below 2 in the high-dose group, which signified a very mild to mild diarrhea condition in that group. In comparison to the model groups that were having a condition of score 4, which represented very severe diarrhea symptoms. Co-treatment or post-treatment of HLJDD did not show significant effect on 5-Fu-induced body weight loss nor diarrhea in mice (Figures 2B,C). The protective effect of pre-treatment of HLJDD was further proven by the histological analysis on intestine structure, which revealed that mice with HLJDD pre-treatment exhibited more normal and intact microscopic intestine structure, while co-treatment or post-treatment of HLJDD can minimally protect intestine tissue from 5-Fu-induced damage (Figure 2D and Table 3). These observations suggested that pre-treatment of HLJDD before using chemotherapeutic agent 5-Fu could reduce undesired side effects.

**TABLE 3:** Overall histological score of jejunum of 5-Fu-treated mice.

Group	Pre-treatment	Co-treatment	Post-treatment
Normal	0.000 ± 0.000	0.000 ± 0.000	0.000 ± 0.000
Model	3.400 ± 0.2828	3.350 ± 0.3536	2.350 ± 0.3536
HLJDD-L	3.450 ± 0.2121	3.350 ± 0.2121	2.250 ± 0.6364
HLJDD-M	2.600 ± 0.2828*	3.000 ± 0.2828	2.050 ± 0.4950
HLJDD-H	1.600 ± 0.1414**	3.050 ± 0.3536	1.900 ± 0.5657

*p < 0.05, **p < 0.01 when compared with the model group.

### Pre-treatment of HLJDD Improved CPT-11-Induced Diarrhea in Mice

CPT-11 is clinically observed to induce frequent diarrhea in patients undergoing chemotherapy of CPT-11-containing regimens. To test if HLJDD can prevent diarrhea induced by CPT-11, we established the CPT-11-induced diarrhea model of mice with some modifications on previously published protocol (Xue et al., 2007). Pre-treatment, co-treatment and post-treatment of HLJDD with CPT-11 were tested and the regimen was illustrated as in Figure 3A. Consistent with 5-Fu, CPT-11 could significantly induce body weight loss in mice. Pre-treatment of HLJDD exhibited potent improvement on the loss of body weight and diarrhea control induced by CPT-11 in a dose dependent manner. Similar to its action in 5-Fu-induced diarrhea model, co-treatment and post-treatment of HLJDD could not achieve body weight gain of CPT-11-treated mice (Figure 3B), nor reverse the situation in CPT-11-induced mice diarrhea (Figure 3C). Histological analysis suggested that pre-treatment but not co-treatment or post-treatment of HLJDD can preserve the microscopic intestine structure of CPT-11-treated mice (Figure 3D and Table 4). These observations suggested that pre-treatment of HLJDD could prevent diarrhea induced by chemotherapeutic agent CPT-11.

**TABLE 4:** Overall histological score of jejunum of CPT-11-treated mice.

Group	Pre-treatment	Co-treatment	Post-treatment
Normal	0.000 ± 0.000	0.000 ± 0.000	0.000 ± 0.000
Model	3.650 ± 0.3536	3.450 ± 0.3536	2.450 ± 0.0707
HLJDD-L	0.3450 ± 0.2121	3.500 ± 0.1414	2.600 ± 0.288
HLJDD-M	2.220 ± 0.2828**	3.350 ± 0.2121	2.050 ± 0.2121
HLJDD-H	1.850 ± 0.0707**	3.650 ± 0.3536	2.000 ± 0.1414

**p < 0.01 when compared with the model group.

### HLJDD Accelerated Intestine Cell Proliferation and Renewal During Chemotherapy-Induced Diarrhea

**FIGURE 4:**
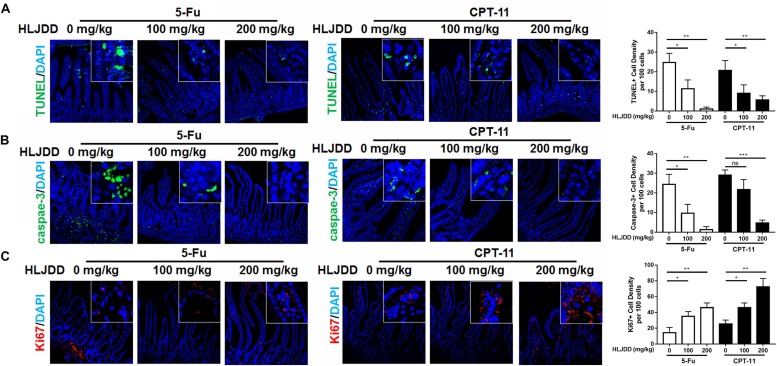
Pre-treatment of HLJDD reduced intestinal cell apoptosis and improved renewal in chemotherapy-treated mice. **(A)** Showed that pre-treatment of HLJDD could significantly reduce the positive TUNEL signal in the intestinal section of chemotherapy-treated mice; **(B)** showed that pre-treatment of HLJDD could significantly reduce the expression of cleaved caspases-3 in the intestinal section of chemotherapy-treated mice; **(C)** showed that pre-treatment of HLJDD could significantly improve the renewal-associated Ki67 expression in the intestinal section of chemotherapy-treated mice. *p < 0.05, **p < 0.01, ***p < 0.001 when compared with the model group.

To further understand the action of pre-treated HLJDD on CID, we applied TUNEL to measure the DNA damage in intestine segments. It was found that both 5-Fu and CPT-11 could significantly induce DNA damage of intestinal cells, as indicated by the presence of TUNEL-positive cells in the intestine segments, while pre-treatment of HLJDD dose-dependently reduce the number of TUNEL-positive cells, suggesting pre-treatment of HLJDD may accelerate the disappearance of damaged cells (Figure 4A). The clearance of damage cells was further evidenced by the observation that less expression of apoptotic marker caspase-3 (Figure 4B). As HLJDD did not directly contact with chemotherapy, we then supposed that the disappearance of apoptotic cells in the intestine segments was possibly attributed by the accelerated replacement of dead cells by newly proliferative cells in the intestine of HLJDD-treated mice. Ki67 was used to stain the newly proliferative cells in the intestine segments. It was shown that pre-treatment of HLJDD could increase the presence of Ki67-positive proliferative cells in the intestine of 5-Fu and CPT-11-treated mice (Figure 4C). These findings suggest that pre-treatment of HLJDD could recover crypt cells in the intestine segments by accelerating its proliferation and renewal, which may contribute to its prevention on CID.

### HLJDD Promoted the Expression of Intestinal Progenitor Cell Markers After Chemotherapy

**FIGURE 5:**
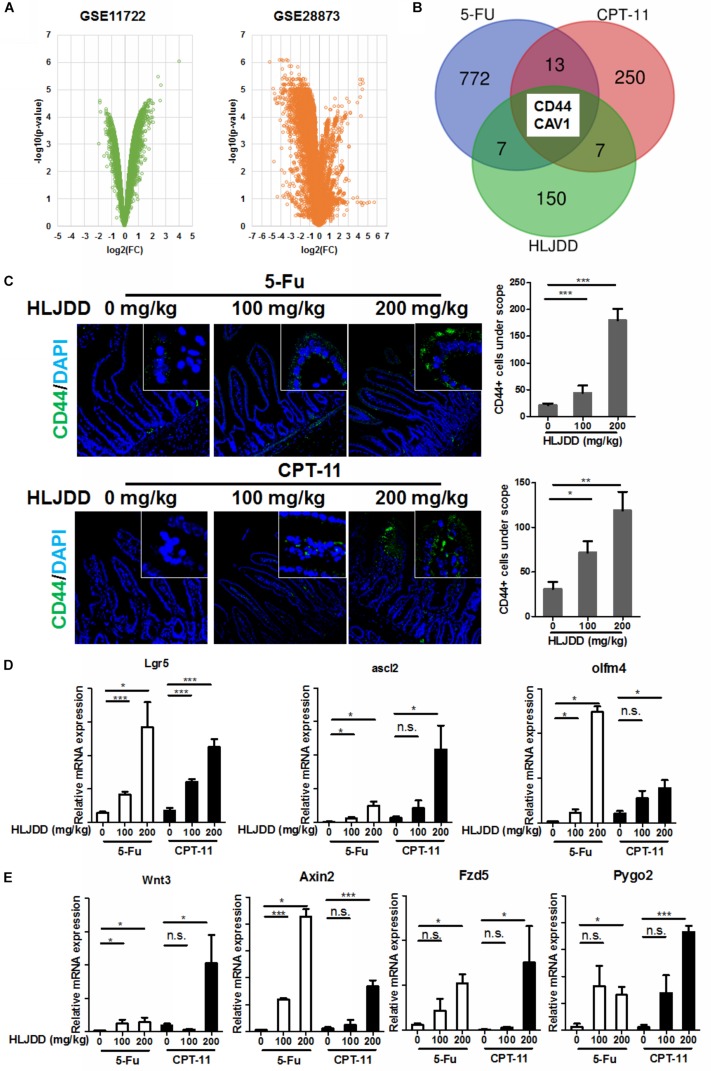
Pre-treatment induced CD44 expression and activation of its downstream Wnt signaling in the intestinal cell of chemotherapy-treated mice. **(A)** showed the volcano plots of gene expression in the intestine challenged by 5-Fu (GSE28873) and CPT-11 (GSE11722). Genes with relative expression of log2(fold change) > 1 and log10(*p*-value) > 2 were shortlisted; **(B)** showed overlapped of genes with significant expression change after 5-Fu and CPT-11 treatment, and genes with expression affected by compounds in HLJDD. Tow genes, CD44 and CAV1, were the common genes among the three populations; **(C)** showed that pre-treatment of HLJDD could significantly improve the CD44 expression in the intestinal section of chemotherapy-treated mice; **(D)** showed that expression of stemness-related gene, including lgr5, ascl2 and oflm4 in the intestine cells in the intestine of chemotherapy-treated mice was induced by pre-treatment of HLJDD; **(E)** showed that expression of Wnt pathway-related genes, including Wnt3, Axin2, Fzd5 and Pygo2, in the intestine cells in the intestine of chemotherapy-treated mice was induced by pre-treatment of HLJDD; *p < 0.05, **p < 0.01, ***p < 0.001 when compared with the model group.

To further understand the mechanism of action, we first identified the genes of intestine cells being regulated commonly by 5-Fu and CPT-11 by accessing the sequencing data in Gene Expression Omnibus (GEO) database. Expression of genes with significant changes after 5-Fu (GSE28873) and CPT-11 (GSE11722) treatment were analyzed and common genes were highlighted (Figure 5A). Prediction of genes being regulated by compounds within HLJDD was extracted from the Comparative Toxicogenomics Database (CTD)^3^. By overlapping the gene lists, we found that CD44 and CAV1 are HLJDD-regulated common genes in 5-Fu and CPT-11-treated intestine. CD44-postive cells in the intestine represent a group of progenitor cells that keeps self-renewal activity and could rapidly differentiate into crypt cells (Figure 5B). We then stained the intestine with antibody to CD44, and observed that pre-treatment of HLJDD could significantly increase the CD44-positive cells at the bottoms of the crypts when mice were exposed to 5-Fu and CPT-11, suggesting that HLJDD pre-treatment may potentiate the progenitor cells in the intestine segments in 5-Fu/CPT-11-treated mice (Figure 5C). This was further proven by the observation that the mRNA expression of stem cell makers such as Lgr5, Ascl2 and Olfm4 were significantly induced in the intestine of HLJDD pre-treated mice (Figure 5D). As CD44 and Lgr5 were reported as the upstream molecules of Wnt/β-catenin signaling (Su et al., 2016; Zhang et al., 2018), we then measured if pre-treatment of HLJDD resulted in activation of Wnt/β-catenin pathway in the intestine segments of 5-Fu/CPT-11-treated mice. Interestingly, in mice without 5-Fu/CPT-11 treatment, HLJDD pre-treatment had no potential effect in elevating the Wnt/β-catenin signaling, but it could significantly induce mRNA expression of Wnt/β-catenin signaling products including Wnt3, Fzd5, Axin2 and Pygo2 (Figure 5E). Our data suggests that HLJDD could promote the repopulation of intestine progenitor cells after chemotherapy probably through activating Wnt/β-catenin signaling.

### HLJDD Improved Tumor Inhibition of Clinical 5-Fu/CPT-11-Containing Chemotherapy Regimens in Colorectal Cancer Model

**FIGURE 6:**
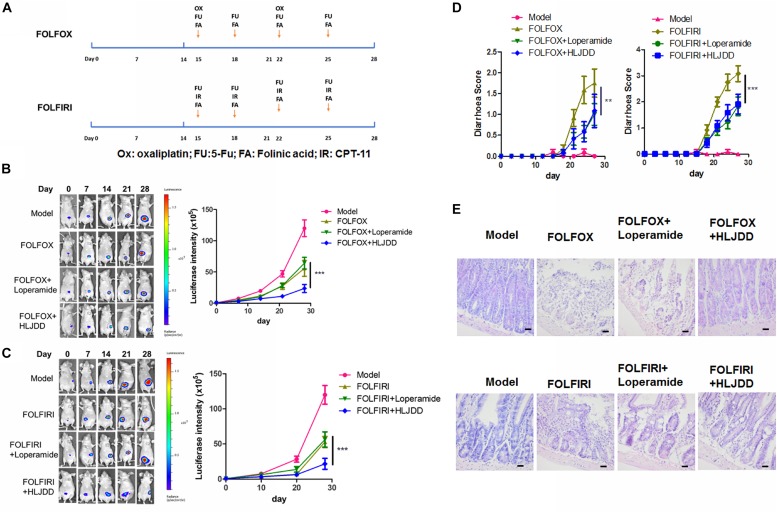
Pre-treatment of HLJDD improved tumor response and diarrhea in clinical chemotherapy regimens FOLFOX- and FOLFIRI-treated colon cancer in mice. **(A)** Showed the regimen plan of FOLFOX and FOLFIRI; **(B,C)** showed that 2-week pretreatment of HLJDD at 200 mg/kg could significantly potentiate the tumor suppressive effect FOLFOX and FOLFIRI on orthotopic colon cancer; **(D)** showed that 2-week pretreatment of HLJDD at 200 mg/kg could significantly improve the diarrhea induced by FOLFOX and FOLFIRI; **(E)** showed that 2-week pretreatment of HLJDD at 200 mg/kg could improve the histological structure of jejunum in FOLFOX- and FOLFIRI-treated colon cancer in mice. **p < 0.01, ***p < 0.001 when compared with the respective chemotherapy regimen group.

5-Fu and CPT-11 are chemotherapeutic agents commonly used in a combing regimen in the clinical treatment of colorectal cancer. Clinical oncologists and physicians designed some common combinations based on the condition of cancer patients, which include FOLFOX and FOLFIRI. To examine if pre-treatment of HLJDD benefits chemotherapy of colorectal cancer in terms of improving treatment outcomes and reducing diarrhea, we established an orthotopic colorectal cancer model in athymic nude mice followed by HLJDD plus FOLFOX/FOLFIRI treatment (Figure 6A). Loperamide, which was a clinically first-line treatment of chemotherapy-induced diarrhea, was introduced as positive control (Andreyev et al., 2014). Significant increase of luciferase signal intensity was observed in control group, indicating that tumor grew fast in the absence of any treatment. FOLFOX or FOLFIRI treatment may reduce the tumor signal in some extent. Loperamide co-treatment have no significant effect on further reducing tumor growth rate in the presence of chemotherapy regimen, while pre-treatment of HLJDD showed further suppression to tumor growth (Figures 6B,C). In addition, Moderate-to-severe diarrhea was observed 3 days after chemotherapeutic regimens started. As the FOLFIRI regimen contains more diarrhea-inducing chemotherapeutic agents (especially CPT-11) than FOLFOX, the earlier initiation of diarrhea was predictable. Co-treatment of loperamide can significantly reduce the diarrhea caused by chemotherapy. Pre-treatment of HLJDD exhibited compatible effect in reducing diarrhea to loperamide, suggesting the potential of HLJDD as a prophylactic treatment to FOLFIRI-induced diarrhea (Figure 6D). Intestine damage was observed in mice of treatment group. While loperamide cannot significantly improve the intestine damage, Mice with HLJDD pre-treatment exhibited improved intestine integrity (Figure 6E and Table 5). These data suggest that HLJDD could enhance the clinical 5-Fu/CPT-11-containing chemotherapy regimens in the treatment of colorectal cancer model via improving treatment outcomes and reducing diarrhea.

**TABLE 5:** Overall histological score of jejunum of FOLFOX- and FOLFIRI-treated mice.

Group	FOLFOX	FOLFIRI
Model	0.5500 ± 0.3536	0.4000 ± 0.1414
Chemotherapy regimen	4.350 ± 0.7778	4.850 ± 0.2121
Loperamide	4.550 ± 0.2121	4.400 ± 0.7071
HLJDD	2.300 ± 0.7071*	2.800 ± 0.4243*

*p < 0.05 when compared with the respective chemotherapy regimen group.

## Discussion

Chemotherapy-induced diarrhea is common in cancer patients under treatment. According to a recent review, the frequency of serious CID (with grading at 3–4) is 5–47% in clinical trials. Grade 1-2 diarrhea is more commonly observed in patients receiving chemotherapeutic agents. Furthermore, some target therapeutic agents and monoclonal antibodies are reported with frequent incidences of diarrhea in patients (Andreyev et al., 2014). Economic burden of CID was estimated to be US$6,600 for every outpatients care of grade 4 diarrhea, according to a recent review based on 22 published articles (Tarricone et al., 2016). CID was observed in 50–80% of patients receiving chemotherapy, which may result in deviation from the planned chemotherapy schedule, leading to chemotherapy failure. Severe CID may be life-threatening (Stein et al., 2010). The diarrhea condition represented by the diarrhea score was significantly alleviated in the HLJDD treated groups, especially in the 200 mg/kg group, from 4 to below 2 (*p* < 0.01). Very promising anti-CID effect was observed by HLJDD in mice model, and it is believed that the same effect could be reproduced with amended dose in human population.

Although CID is frequently observed as an established gastrointestinal side effect of cancer chemotherapy, there is few studies trying to understand its pathological mechanisms. It was suggested that changes of gut microbiota and secretion of mucin would result in CID (Stringer et al., 2007; Li et al., 2017). It was also claimed that CID could be a result of intestinal mucosa damage, which repeatedly trigger apoptotic and inflammatory events in intestinal epithelium and bowel wall (Lee et al., 2014). Notably, some recent studies have highlighted the role of intestinal progenitor cells in the crypts in the regeneration and integrity of intestinal epithelium. Due to the rapid renewal property, intestinal progenitor cells are easily but non-specifically targeted by the chemotherapeutic agents that aim to kill fast growing cancer cells. Previous studies have suggested that chemotherapeutic agents could induce apoptosis and inhibit proliferation of intestinal progenitor cells (Dekaney et al., 2009; Zhan et al., 2014). In our study, we observed that pre-treatment of HLJDD could significantly preserve intestinal progenitor cells in crypts of mice receiving chemotherapy by a notable increase in CD44+ cells under scope (*p* < 0.01). Given that HLJDD was not given simultaneously with chemotherapeutic agents, it is not possible that HLJDD renders any reduced absorption or abortion from the cells. Instead, HLJDD recovered the Wnt/β-catenin signaling activity in the CD44+ intestinal progenitor cells in the crypts of mice receiving chemotherapy (*p* < 0.05). Interestingly, HLJDD did not elevate the basic level of Wnt/β-catenin signaling in mice without chemotherapy but can sustain its activity during chemotherapy. Considering the continuous activation of Wnt/β-catenin signaling would be a risk of tumorigenesis (Joosten et al., 2017), HLJDD might not likely to expose the patients who are going to receive chemotherapy under a high risk of tumorigenesis. An unenviable confounding variable here was that the pre-treatment of HLJDD was given at the early stage of tumor development, while the co-treatment and post-treatment were given at relatively later stages. There could be possibly differences in action of HLJDD on the intestine.

5-Fu and CPT-11 are given systemically for anal, breast, colorectal, oesophageal, stomach, pancreatic and skin cancers. An early study of the efficacy and toxicity of irinotecan for patients with metastatic colorectal cancer reported that, approximately 56% of patients receiving CPT-11 demonstrated symptoms of diarrhea (grade 3-4) (Conti et al., 1996). Lenfers et al. (1999) even postulated as high as 86% of grade 3-4 diarrhea in patients receiving CPT-11 and 57% in patients with 5-FU. Combination treatment of leucovorin, 5-FU and CPT-11 showed improvement in survival and response rate of metastatic colorectal cancer patients, however, the incidence rate of grade 3 diarrhea is 13.2% and grade 4 diarrhea is approximately 7% (Saltz et al., 2000). There are not many publications directly studying the chemo-regimens FOLFOX and FOLFIRI in animal model. According to the few papers regarding the use of chemotherapeutic regimens in animal (Robinson et al., 2013a, b), we designed and adjusted the dose and treatment frequency of chemotherapeutic agents. Using the orthotopic colorectal cancer model, we validated that the modified FOLFOX and FOLFIRI can well mimic the clinical efficacy and intestine toxicity in patients. The successful translation of FOLFOX and FOLFIRI regimen from clinical setting to animal model would offer a good reference for the further studies of strategies in improving chemotherapy in colorectal cancers.

Official guideline of management of CID is yet available, though some tentative protocols have been recommended based on literature review by multidisciplinary team (Andreyev et al., 2014). Current clinical treatment of CID includes loperamide and octreotide. Administration of loperamide remains to be the first line treatment and subcutaneous injection of octreotide together with antibiotics will be intervened for patients who failed high doses of loperamide and developed grade 3-4 symptoms (Benson et al., 2004). In our study, we tested loperamide as a positive control to stop diarrhea in colorectal cancer mice treated with chemotherapy regimens. Although it could significantly prevent the occurrence of diarrhea during chemotherapy, loperamide is not likely beneficial to the tumor regression by FOLFOX or FOLFIRI given its mechanism of antidiarrheal effect. Interestingly, the tumor growth was restricted during HLJDD treatment, indicating that the synergistic effect of HLJDD may be the result of its prophylactic control before patients go into chemotherapeutic regimen. In addition, pre-treatment of HLJDD can significantly alleviate diarrhea and intestine damage induced by chemotherapeutic regimens. Considering its efficacy and safety, HLJDD may be potentially an adjuvant treatment to chemically relevant treatments of colorectal cancer.

With regards to the long history of HLJDD usage in traditional Chinese medicine for thousands of years, the safety of this prescription on patient is unquestionably assured. Before the clinical application of HLJDD to reduce the side effects as well as enhance tumor response with chemotherapy such as 5FU and CPT-11, its efficacy on human population should be justified in human clinical trial. A randomized double-blind controlled study to compare the effect of HLJDD to a placebo could be an option. Notably, although HLJDD was experimentally demonstrated to be effective on reducing CID, more clinical data reporting benefits in future study are needed before application as adjuvant to cancer treatment in patients. Every action of intaking medicine should be consulted by practitioners.

## Conclusion

We systemically evaluated the potential of a Chinese herbal formula HLJDD as prophylactic treatment of CID. Quality and stability of the herbal extract of HLJDD was assessed by chemical fingerprinting, and no apparent degradation of major active compounds in the decoction after 24-month storage was shown. Pre-treatment but not co- or post-treatment of HLJDD could dose-dependently prevent body weight loss, diarrhea and intestinal damage induced by chemotherapeutic agents 5-Fu and CPT-11. This effect of HLJDD has been correlated with reduced intestinal cell apoptosis and improvement of cell renewal. Target identification suggested that CD44 level in renewing crypt cells could be maintained by HLJDD pre-treatment, and restoration of CD44 improved certain level of Wnt-signaling pathway activity to maintain the rapid cell renewal for repairing the intestinal wall during chemotherapy. In addition, pre-treatment of HLJDD improved the efficacy of 5-Fu and CPT-11-containing chemotherapeutic regimens FOLFOX and FOLFIRI in suppressing orthotopic tumor growth of human colorectal cancer. Our study sheds light on the potential of HLJDD as a neoadjuvant treatment for chemotherapy by improving diarrhea and tumor response.

## Data Availability Statement

Publicly available datasets were analyzed in this study, these can be found in the Comparative Toxicogenomics Database (CTD, http://ctdbase.org/); the NCBI Gene Expression Omnibus (GEO) database (GSE28873 and GSE11722).

## Ethics Statement

The animal study was reviewed and approved by Committee on the Use of Live Animals in Teaching and Research (CULATR) of the University of Hong Kong.

## Author Contributions

YF and NW designed and conceived the study. Y-TC, CZ, H-YT, BF, and NW did experiments and analyzed the data. HN provided standardized extract of HLJDD. Y-TC, FC, NW, and YF drafted the manuscript. All authors revised and confirmed the manuscript.

## Conflict of Interest

HN was employed by the company PuraPharm International (H.K.) Ltd.

The remaining authors declare that the research was conducted in the absence of any commercial or financial relationships that could be construed as a potential conflict of interest.

## Footnotes

1 http://ctdbase.org/

2 http://bioinformatics.psb.ugent.be/webtools/Venn/

3 http://ctdbase.org/
